# Costs of orphan medicinal products: longitudinal analysis of expenditure in Wales

**DOI:** 10.1186/s13023-023-02956-3

**Published:** 2023-11-01

**Authors:** Yankier Pijeira Perez, Eifiona Wood, Dyfrig A Hughes

**Affiliations:** Centre for Health Economics and Medicines Evaluation, Bangor University, Ardudwy, Normal Site, Holyhead Road, Bangor, Gwynedd, Wales LL57 2PZ UK

**Keywords:** Orphan Drugs, Drug costs, Costs analysis, health economics

## Abstract

**Background:**

The Orphan Regulation ((EC) No 141/2000) has successfully redirected private and public investment towards previously neglected areas through incentives, regulatory obligations and rewards. However, the growth in the number of licensed orphan medicinal products (OMPs) has led to concerns about increased costs. The aims were to investigate the trend in the costs of OMPs to the National Health Service in Wales, to attribute costs of medicines within and outside periods of marketing exclusivity, and estimate the contribution of individual medicines to the overall costs of OMPs.

**Methods:**

Expenditure on OMPs in Wales was analysed between the 2014/15 and 2019/20 financial years using data on prescriptions dispensed in primary care, secondary care, and specialised commissioned services. OMP spend was calculated as a proportion of total medicines expenditure, whether it was incurred during, or outside the marketing exclusivity period (MEP), and by therapeutic area and medicine.

**Results:**

Overall spend on OMPs and all medicines increased from £32 m to £82 m, and from £1,030 m to £1,198 m, respectively, with the proportion of spend on OMPs more than doubling from 3.1% to 6.9% per annum. Average year-on-year growth in the costs of OMPs was 21%, compared to 2% for other medicines. Costs following MEP expiry contributed significantly to overall OMP costs, increasing from £8 m to £30 m, corresponding to an increase from 24% to 37%. Treatments for ‘malignant disease and immunosuppression’, ‘nutrition and blood’ and the ‘respiratory system’ accounted for 90% of all OMP spend. Half of total OMP annual expenditure was on just 4 medicines in 2014/15, increasing to 8 in 2019/20.

**Conclusions:**

Both the number of OMPs and the amount spent on OMPs in Wales has increased over time, possibly as a consequence of favourable licensing conditions, permissive health technology assessment policies and dedicated funding.

## Introduction

Favourable European regulatory conditions have made the orphan medicinal product (OMP) market an attractive prospect. This is evidenced by the increasing numbers of OMPs authorised over the two decades since the regulations came into effect [1]. By September 2021, the European Commission had designated 1,824 promising drugs with orphan status and authorised 204 for marketing. Indeed, the proportion of novel authorised medicines for rare disease indications appears to be disproportionately high relative to the burden of prevalent chronic diseases. In 2019, for instance, seven of the 30 new active substances recommended for marketing authorisation by the European Medicines Agency were OMPs [2].

The small markets associated with rare diseases typically result in higher drug prices [3] which, together with weaker clinical evidence compared to non-orphan medicines [4], has led to concerns about their cost-effectiveness, and has impacted on patient access across many European countries [5–7]. Decision-making bodies are consequently challenged by the quandary of making high-cost medicines available to treat few individuals often with incremental cost-effectiveness ratios that exceed thresholds for approval [8–10]. However, health technology and reimbursement organisations are generally cognisant that a strict utilitarian approach, which promotes the greatest gain for the greatest number of patients, would introduce inequity of access for patients with rare conditions [11–13].

These factors combined has led to a steady but significant upward trend in the availability, prescribing and therefore total costs of OMPs internationally. Added to which are concerns that OMPs are associated with higher returns on investment and profitability for their manufacturers [14]. An analysis from 2010 projected that the costs of OMPs across Europe, as a proportion of total pharmaceutical expenditure, would be expected to plateau in the decade to 2020 [15], but expenditure has in fact increased at a compound annual growth rate of 16% over this period [16]. The costs to European Union member states’ health systems for reimbursing OMPs between 2000 and 2017 totalled about €20–25 billion [17]. More detailed evidence from individual countries corroborate the observed increases in costs. In Sweden and France, for instance, OMPs represented 2.7% and 3.2% of total pharmaceutical expenditure, respectively, in 2013 [18]; and in Bulgaria, between 2014 and 2016, OMPs represented 9% of the National Health Insurance Fund spend on medicines [19]. Cost pressures have been exacerbated by a lack of generic competition upon expiry of market exclusivity. Of 70 medicines with expired orphan status, less than 20% faced generic or biosimilar competition [20].

Within the UK, the publicly funded National Health Service (NHS) provides comprehensive healthcare to all its citizens. The service is administered separately by each constituent country. NHS Wales serves 3.1 million people and is organised into 7 health boards which provide emergency services and a range of primary, secondary, and specialist tertiary care. Health boards have a legal requirement to make available, within 60 days, medicines that have been recommended for use in the NHS by the National Institute for Health and Care Excellence (NICE) and the All Wales Medicines Strategy Group (AWMSG) [21]. The £80 million New Treatments Fund for Wales [22] meets the costs of new medicines for the first year, after which health boards are obliged to fund treatments within existing budgets. Both NICE and the AWMSG operate policies that provide more flexibility around the decision-making parameters for highly specialised technologies and “ultra-orphan” medicines, which account for a subset of OMPs used to treat diseases with a prevalence of ≤ 1 per 50,000 [21, 23]. For the period between 2002 and 2014, orphan and ultra-orphan medicinal products were less likely to be approved than non-orphan medicinal products (59% and 73% versus 81%, respectively) [24]; whereas following the introduction of the AWMSG rare diseases policy in 2015, all 9 OMPs, and 5 of the 7 ultra-orphans were recommended for use in NHS Wales [25].

The aim of the present analysis was to investigate the trend in the costs of OMPs to NHS Wales, categorised by broad therapeutic areas and according to expenditure against primary, secondary, or specialist commissioned budgets. Furthermore, the analysis attributed costs to medicines within and outside periods of marketing exclusivity and identified the contribution of individual OMPs to the overall costs.

## Methods

Data on primary care prescribing of medicines between the 2014/15 and 2019/20 financial years were obtained from the Comparative Analysis System for Prescribing Audit (CASPA) database, which records all prescriptions issued outside of hospitals in Wales. Secondary care expenditure on medicines was based on hospital pharmacy summary dispensing data and obtained from the Medicine Usage (Medusa) database for the financial periods 2009/10 to 2019/20. Data on the cost of specialist commissioned medicines between 2014/15 and 2019/20 were obtained directly from the Welsh Health Specialised Services Committee (WHSSC), which is responsible for the planning of specialised and tertiary services on behalf of Health Boards. These three data sources provided a complete, nationwide compilation of expenditure on all medicines in Wales.

OMPs were identified from the European Medicines Agency [26] and cross-checked against the Orphanet database [27]. Medicines that were used to treat rare diseases prior to 2000, when new legislation [28] for designation of orphan medicines was introduced, and those without formal orphan drug status were not included. Authorisation and expiry dates based on the market exclusivity period (MEP) of each orphan medicine were recorded to ensure correct identification of orphan status as of 30 June 2020 [26]. The orphan indication(s) of each medicine was noted and categorised according to legacy chapters of the British National Formulary (BNF) [29]. This is a broad classification based on organ/system/disease that was used widely in the UK until 2017.

Expenditure data from the primary care, secondary care and specialised services datasets were matched with the OMP dataset. In order to determine which medicines to include as OMPs, a variety of approaches were applied to ensure appropriate categorisation of the medicinal product as orphan or non-orphan. Firstly, branded OMPs were cross-matched and included. Secondly, medicines listed by generic name were identified on a case by case basis according to the information available in the datasets and assessed for OMP status. This included a review of BNF codes for primary care data, hospital department cost codes for secondary care data (e.g. for differentiating a paediatric orphan formulation from an adult non-orphan listing), and formulation type (e.g. distinguishing an orphan medicine eye-drop formulation and a non-orphan non-eye-drop formulation).

Data were analysed descriptively to compare the total NHS Wales expenditure on OMPs (the sum of CASPA, MEDUSA and WHSSC figures) with total pharmaceutical expenditure, in terms of absolute and relative increase over time. Additional analyses considered orphan drug expenditure by broad therapeutic area and the contribution of individual orphan products to the total costs of OMPs.

All the data were analysed using RStudio Version 1.3.1093, © 2009–2020 RStudio, PBC.

## Results

The number of OMPs with marketing approval has increased steadily, from 60 medicines for 83 indications in 2009/10 to 115 medicines for 147 indications by 2019/20.

### Medicines expenditure

**Fig. 1:**
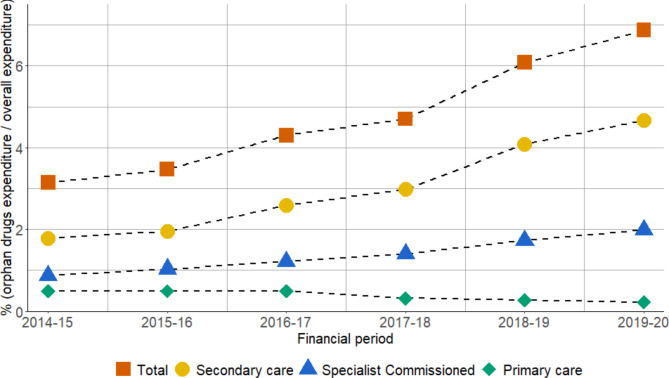
Orphan medicinal product expenditure between 2014/15 and 2019/20 as a percentage of the total spend on medicines, and by prescribing source

For the period where complete data were available, between 2014/15 and 2019/20, the total expenditure on all medicines in Wales increased from £1.031 billion to £1.198 billion, whilst OMP expenditure increased from £32 million to £82 million (Table 1). As a proportion of total pharmaceutical spend, expenditure on OMP increased from 3.1% to 6.9% per annum during this period (Fig. 1). Overall growth in medicines expenditure was 16%, yet growth observed in non-OMPs was less, at 12%, compared with OMPs which grew by 154%. Average year-on-year growth for all non-OMPs was 2%, compared to 21% for OMPs.

**Table 1:** NHS Wales expenditure on all medicines and orphan medicinal products, by source and financial period

Financial period	Total expenditure (£, millions)				Orphan expenditure (£, millions)			
	Secondary Care	Primary Care	Specialist Commissioned	Total	Secondary Care	Primary Care	Specialist Commissioned	Total
2009/10	209.00	-	-	209.00	8.74	-	-	8.74
2010/11	226.62	-	-	226.62	9.52	-	-	9.52
2011/12	241.06	-	-	241.06	10.97	-	-	10.97
2012/13	250.63	-	-	250.63	12.19	-	-	12.19
2013/14	275.20	-	-	275.20	14.57	-	-	14.57
2014/15	293.63	727.87	9.21	1,030.70	18.34	5.03	9.00	32.37
2015/16	322.82	744.23	11.51	1,078.56	21.05	5.24	11.13	37.42
2016/17	363.52	724.11	13.71	1,101.34	28.57	5.40	13.37	47.34
2017/18	370.13	726.27	15.82	1,112.22	33.30	3.46	15.56	52.33
2018/19	394.46	709.92	19.62	1,124.00	45.87	3.01	19.48	68.35
2019/20	427.12	747.51	23.85	1,198.48	55.93	2.69	23.71	82.33

(-) Expenditure data from CASPA and WHSSC for primary care and specialist commissioned medicines were not available prior to 2014/15

The majority of OMP expenditure was observed in secondary care and specialist commissioned healthcare. Between 2014/15 and 2019/20, total secondary care expenditure on OMP (both within and outside their MEP) increased from £18.3 million to £55.9 million, corresponding to an increase from 6% to 13% of secondary care medicine spend. Expenditure on all medicines in primary care remained static (upper and lower range between 2014/15 and 2019/20: £728 million to £748 million) with OMPs accounting for a reducing proportion of overall spend, from £5.0 million (0.7%) in 2014/15 to £2.7 million (0.4%) in 2019/20 (Table 1). Expenditure on pharmaceutical spend in specialised healthcare services more than doubled between 2014/15 and 2019/20 (from £9 million to £24 million) of which nearly all (98%) was accounted for by OMPs.

### End of marketing exclusivity period

Expenditure on OMPs increased over time, regardless of marketing exclusivity status (Table 2). Between 2014/15 and 2019/20, the number of OMPs within their marketing exclusivity period increased from 57 to 72 whilst the number of those which were beyond this period quadrupled from 11 to 44. Whilst total spend within MEP remained higher than outside MEP, spend on OMP once exclusivity had expired increased from £7.9 million in 2014/15 to £30.5 million in 2019/20, accounting for an increasing proportion of orphan drug spend, from 24 to 37% during this period; this increase is driven by notably higher expenditure outside of MEP since 2018/19.

**Table 2:** Orphan medicinal product expenditure within and outside marketing exclusivity period

	Total spend, £, million (number of unique OMPs)							
	Secondary Care		Primary Care		Specialist Commissioned		Total spend	
Financial period	Within MEP	Outside MEP	Within MEP	Outside MEP	Within MEP	Outside MEP	Within MEP	Outside MEP
2009/10	6.76 (41)	1.98 (1)					6.76 (41)	1.98 (1)
2010/11	7.05 (43)	2.47 (1)					7.05 (43)	2.47 (1)
2011/12	8.47 (45)	2.50 (2)					8.47 (45)	2.50 (2)
2012/13	10.16 (46)	2.03 (4)					10.16 (46)	2.03 (4)
2013/14	11.21 (44)	3.36 (8)					11.21 (44)	3.36 (8)
2014/15	14.92 (47)	3.42 (9)	1.80 (21)	3.23 (3)	7.74 (26)	1.26 (6)	24.46 (57)	7.91 (11)
2015/16	17.27 (47)	3.78 (13)	1.89 (21)	3.35 (6)	9.39 (31)	1.74 (7)	28.55 (60)	8.87 (16)
2016/17	23.20 (55)	5.37 (17)	2.08 (23)	3.32 (9)	11.29 (32)	2.08 (11)	36.57 (67)	10.77 (21)
2017/18	27.07 (57)	6.24 (23)	1.47 (19)	1.99 (11)	13.26 (36)	2.30 (14)	41.80 (69)	10.53 (27)
2018/19	33.86 (61)	12.01 (30)	1.34 (15)	1.66 (14)	9.85 (33)	9.63 (13)	45.05 (70)	23.30 (35)
2019/20	37.48 (64)	18.45 (39)	1.22 (15)	1.47 (15)	13.18 (38)	10.53 (16)	51.88 (72)	30.45 (44)

(-) CASPA and WHSSC data not available prior to 2015/15

MEP marketing exclusivity period

### Expenditure by BNF chapter

**Fig. 2:**
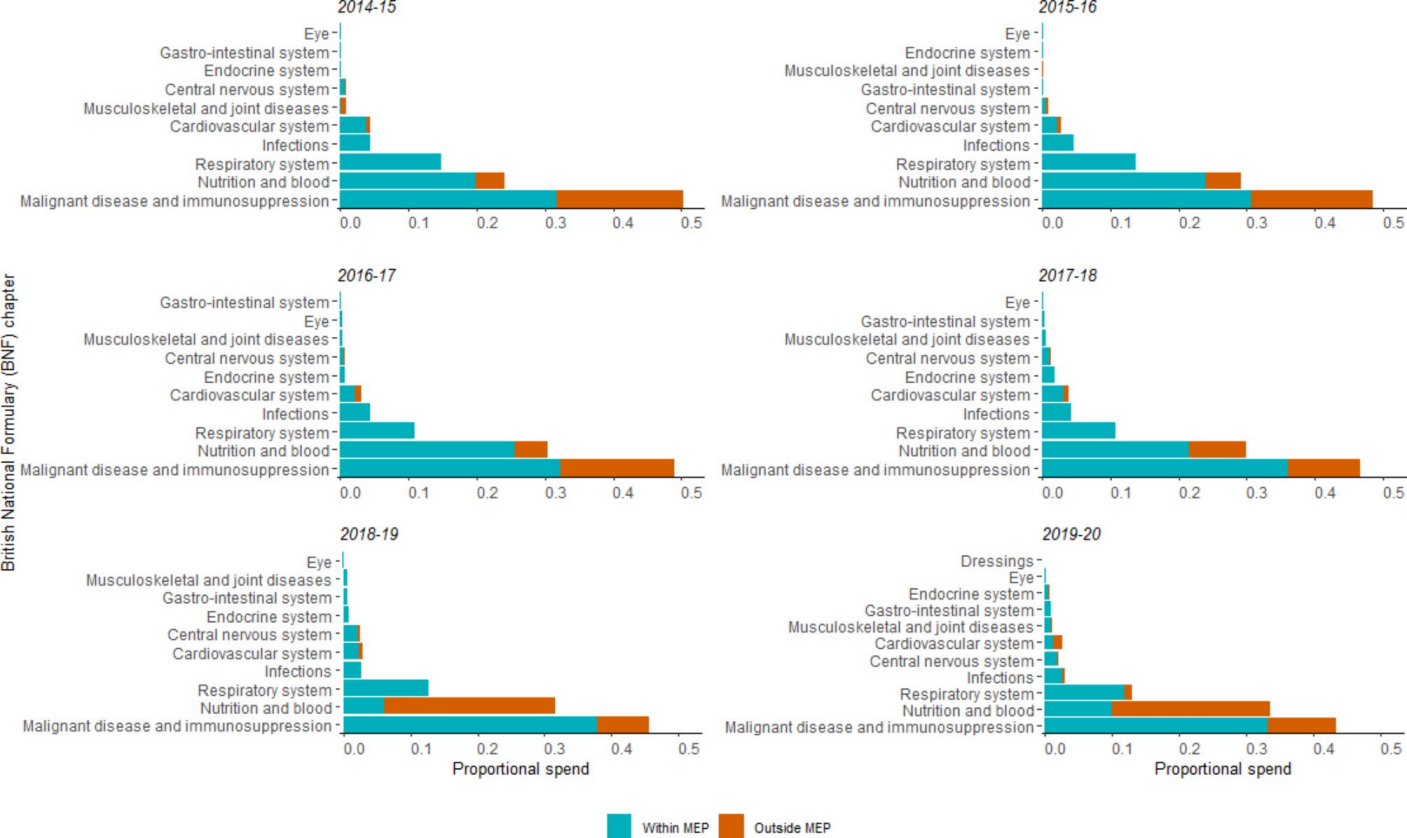
Proportion of orphan medicinal product expenditure by marketing exclusivity status, BNF category, and by financial period

For the period 2014/15 to 2019/20, 90% of OMP spend could be attributed to three disease areas—£148.9 million (47%) attributed to ‘malignant disease and immunosuppression’, £98.0 million (31%) to ‘nutrition and blood’, and £40.2 million (13%) to ‘respiratory’ (Fig. 2). The proportion of orphan drug spend by BNF chapter during this period also increased, from 15% to 21% for ‘malignant disease and immunosuppression’, and from 11% to 28% for ‘nutrition and blood’.

### Expenditure by OMP

**Fig. 3:**
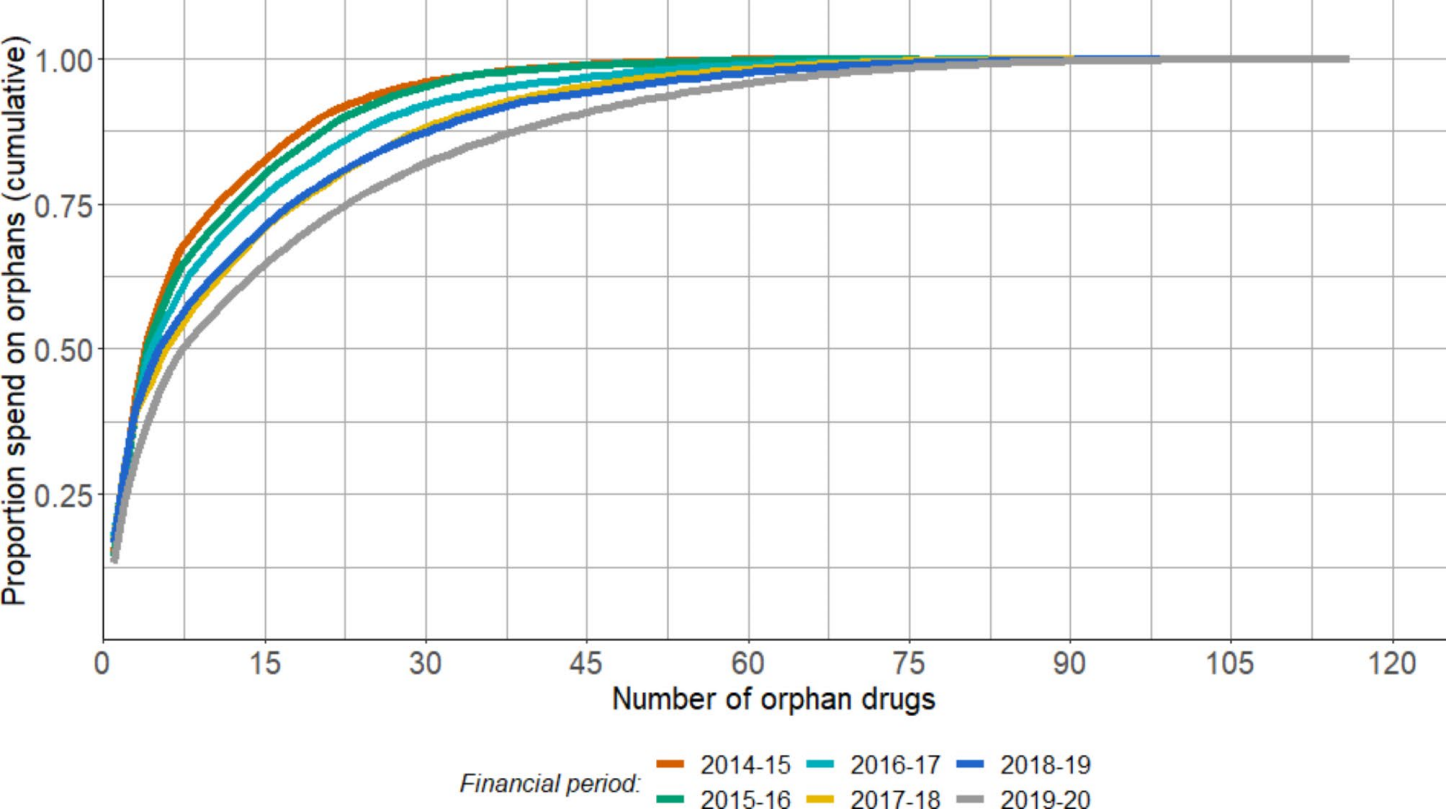
Cumulative contribution of orphan medicinal product expenditure, by financial period

Four medicines accounted for 50% of total OMP annual expenditure in 2014/15 and 2015/16. This increased to 5 in 2016/17, 6 in both 2017/18 and 2018/19, and 8 medicines in 2019/20 (Fig. 3). Since 2014/15, 60% of total OMP spend can be attributed to 10 medicines—eculizumab, lenalidomide, ivacaftor, imatinib, ibrutinib, nilotinib, azacitidine, sunitinib, daratumumab and romiplostim (Table 3). Of these, seven fall under the BNF ‘malignant disease and immunosuppression’ category, two (eculizumab and romiplostim) are used to treat ‘nutrition and blood’ disorders and one (ivacaftor) is for the ‘respiratory system’.

**Table 3:** Expenditure on the top 10 orphan medicinal products (by total cost) for the periods 2009/10–2019/20, and 2014/15–2019/20

Expenditure 2009/10 to 2019/20^a^			Expenditure 2014/15 to 2019/20		
Orphan medicine	Cost (£)	(% total orphan spend)	Orphan medicine	Cost (£)	(% total orphan spend)
lenalidomide	49,997,279	13.29	eculizumab	48,037,137	15.00
eculizumab	48,060,011	12.78	lenalidomide	36,455,658	11.39
ivacaftor	34,026,894	9.05	ivacaftor	32,887,314	10.27
imatinib mesilate	26,171,612	6.96	imatinib mesilate	18,411,191	5.75
sunitinib malate	18,899,422	5.02	ibrutinib	12,053,813	3.77
nilotinib	13,092,369	3.48	nilotinib	11,401,227	3.56
azacitidine	12,976,966	3.45	azacitidine	9,922,398	3.10
ibrutinib	12,053,813	3.20	sunitinib malate	8,376,401	2.62
romiplostim	8,052,131	2.14	daratumumab	7,614,869	2.38
daratumumab	7,614,869	2.02	romiplostim	6,659,175	2.08

^a^ CASPA and WHSSC data not available prior to 2014/15

## Discussion

Over the period of observation, the number of OMPs approved for marketing by the European Medicines Agency has increased steadily, with a corresponding year-on-year increase in expenditure by NHS Wales. Within the time period for which the complete dataset was available, between 2014/15 and 2019/20, the proportion of spend on OMP increased from 3% to 7% of all expenditure on pharmaceutical products. The year-on-year growth in expenditure on OMPs was considerably higher than non-OMP (21% vs. 2%). OMP expenditure is primarily associated with medicines categorised for the treatment of ‘malignant disease and immunosuppression’, ‘nutrition and blood’ and the ‘respiratory system’. Of note, however, is the skewness of costs, with more than half of total orphan drug expenditure being attributed to just 4 medicines (increasing to 8 by 2019/20). Expenditure on these products increased both in absolute terms, and notably so as a proportion of total spend on OMP since 2018/19, in the period following expiration of MEP.

Our findings are consistent with an analysis across eight European countries which identified that the OMP share of total pharmaceutical expenditure has increased each year since 2000, rising to 7.2% in 2017 [16]. In Wales, we found this increase to occur predominantly in secondary care and specialist commissioned services and could be attributed to the high prices of treatments for specialist populations, alongside favourable regulatory conditions, HTA processes that allow for more flexibility in approving orphan medications and dedicated funding to improve short-term access. Earlier projections, indicating that the costs of orphan drugs across Europe would reach a peak of 4.6% in 2016 followed by a levelling off to between 4% and 5% to 2020 [15], have not been observed in Wales; although a later analysis of OMP expenditure across 8 European countries indicated that the relative spending on OMPs has increased over the last 20 years [16]. These percentages are, of course, also dependent on the growth in non-OMP costs.

Our analysis has strengths in considering all prescription medicines issued in Wales, at least for the financial years 2014/15 to 2019/20. However, there were limitations, most notably the absence of data on prescribing volumes. While total costs are a function of the number of approved OMP and their respective clinical indications, volume of use and unit prices, we could only be confident in the small contribution of unit price variations to changes in overall costs. An analysis of volume might have also assisted in identifying trends in prescribing habits of non-OMPs relative to OMPs, and any changes in clinical practice as a result of the increasing availability of OMPs.

The expenditure data supporting the analysis was based on hospital prices which are subject to purchasing agreements and may differ to the published list price. Furthermore, rebates associated with patient access schemes that are subsequently reimbursed by manufacturers were not captured in the analysis. Given this, and the confidential nature of the pricing agreements between NHS Wales and suppliers which may include discounts, rebates and risk-sharing schemes, a pragmatic approach to not take inflation into account was followed, but this could also be considered a limitation within the analysis.

Finally, the costing method required identification of certain OMPs by reference to specific cost centres, such as those with an alternative, non-orphan indication. This approach is reliant on accurate cost centre charging by pharmacies and consequently may be subject to a small margin of error but is unlikely to meaningfully impact our findings.

The continued high cost of OMPs beyond the MEP has been a driver for the continued year-on-year increase in their costs, linked to the high barriers to market entry for competitor generic or biosimilar medicines [20]. While there is recognition that the 10-year market exclusivity period may not be fully justified for certain OMPs [17], it would require reform of the orphan drugs legislation to promote competition upon expiry of MEP to facilitate a reduction in prices. It is unclear whether the planned revisions to the Orphan Regulation (Regulation (EC) No 141/2000) [30] will be transposed into UK law.

Our research findings, that prescribing is becoming increasingly directed towards treating rare conditions (defined by a prevalence of ≤ 1 in 2,000) with expensive treatments, corresponds with the findings of Mestre-Ferrandiz et al. [16]. Whether this current trend continues, in light of changes to the regulatory landscape in the UK [31], and is sustainable, especially in the context of orphan advanced therapy medicinal products, remains to be seen. Nonetheless it relates to the greater flexibility in HTA policies for rare disease medicines [32], with dedicated funding aimed to promote rapid access resulting in medicines benefiting a few patients accounting for a growing proportion of spend on pharmaceutical products overall [33]. This has implications in the wider debate around societal preferences for the expensive treatment of rare diseases in relation to the notion of maximising health production [34, 35].

## Conclusions

This first, comprehensive analysis of orphan medicine expenditure in a UK setting identified that both the number of OMPs and the amount spent on OMPs has increased over time, primarily associated with new treatments for malignancies and haematological conditions. This trend may be attributed to the introduction of favourable licensing conditions afforded to OMP as well as the rising costs associated with highly specialised treatments.

## Data Availability

Data on orphan medicinal products are available from resources available in the public domain, and which are referenced in the article. Data on NHS Wales expenditure on orphan and all medicinal products are available from https://whssc.nhs.wales and https://dhcw.nhs.wales subject to third part restrictions.
